# SARS-CoV-2 Omicron variants BA.4 and BA.5 dominated the fifth COVID-19 epidemiological wave in Mexico

**DOI:** 10.1099/mgen.0.001120

**Published:** 2023-12-19

**Authors:** Blanca Itzelt Taboada, Selene Zárate, Rodrigo García-López, José Esteban Muñoz-Medina, Bruno Gómez-Gil, Alfredo Herrera-Estrella, Alejandro Sanchez-Flores, Angel Gustavo Salas-Lais, Benjamin Roche, Gabriela Martínez-Morales, Hermilo Domínguez Zárate, Célida Duque Molina, Ricardo Avilés Hernández, Susana López, Carlos F. Arias

**Affiliations:** ^1^​ Departamento de Genética del Desarrollo y Fisiología Molecular, Instituto de Biotecnología, Universidad Nacional Autónoma de México, Cuernavaca 62210, Mexico; ^2^​ Posgrado en Ciencias Genómicas, Universidad Autónoma de la Ciudad de México, Mexico City 03100, Mexico; ^3^​ Coordinación de Calidad de Insumos y Laboratorios Especializados, Instituto Mexicano del Seguro Social, Mexico City 07760, Mexico; ^4^​ Centro de Investigación en Alimentación y Desarrollo AC, Coordinación Regional Mazatlán, Acuicultura y Manejo Ambiental, Mazatlan 82100, Mexico; ^5^​ Laboratorio Nacional de Genómica Para la Biodiversidad-Unidad de Genómica Avanzada, Centro de Investigación y de Estudios Avanzados del IPN, Irapuato 36824, Mexico; ^6^​ Unidad Universitaria de Secuenciación Masiva y Bioinformática, Instituto de Biotecnología, Universidad Nacional Autónoma de México, Cuernavaca 62210, Mexico; ^7^​ Laboratorio Central de Epidemiología, Instituto Mexicano del Seguro Social, Mexico City 02990, Mexico; ^8^​ Infectious Diseases: Vector, Control, Genetic, Ecology and Evolution (MIVEGEC) Université de Montpellier, IRD, CNRS, 34090 Montpellier, France; ^9^​ Dirección de Prestaciones Médicas, Instituto Mexicano del Seguro Social, Ciudad de México 06700, Mexico; ^10^​ Unidad de Planeación e Innovación en Salud, Instituto Mexicano del Seguro Social, Ciudad de México, Mexico

**Keywords:** emerging sublineages, genomic surveillance, Mexico, Omicron, SARS-COV-2

## Abstract

In Mexico, the BA.4 and BA.5 Omicron variants dominated the fifth epidemic wave (summer 2022), superseding BA.2, which had circulated during the inter-wave period. The present study uses genome sequencing and statistical and phylogenetic analyses to examine these variants' abundance, distribution, and genetic diversity in Mexico from April to August 2022. Over 35 % of the sequenced genomes in this period corresponded to the BA.2 variant, 8 % to the BA.4 and 56 % to the BA.5 variant. Multiple subvariants were identified, but the most abundant, BA.2.9, BA.2.12.1, BA.5.1, BA.5.2, BA.5.2.1 and BA.4.1, circulated across the entire country, not forming geographical clusters. Contrastingly, other subvariants exhibited a geographically restricted distribution, most notably in the Southeast region, which showed a distinct subvariant dynamic. This study supports previous results showing that this region may be a significant entry point and contributed to introducing and evolving novel variants in Mexico. Furthermore, a differential distribution was observed for certain subvariants among specific States through time, which may have contributed to the overall increased diversity observed during this wave compared to the previous ones. This study highlights the importance of sustaining genomic surveillance to identify novel variants that may impact public health.

## Data Summary

All 7022 SARS-CoV-2 sequence data generated in this work have been deposited to the Global Initiative on Sharing All Influenza Data (GISAID, https://www.gisaid.org/) with the accession EPI_SET_230621bg and to GenBank. The accession number and metadata for all these sequences are provided in Table S1, available in the online version of this article, which also contains the corresponding GenBank accession numbers. We acknowledge the other laboratory-generated genomes by providing GISAID acknowledgement tables in Table S2 that include a snapshot of the data provided along a hyperlink to access the contributors of each individual sequence with details such as accession number, virus name, collection date, originating laboratory, submitting laboratory and the list of authors. Epidemiological data of Mexico was obtained through the Secretaría de Salud website (https://www.gob.mx/salud/documentos/datos-abiertos-152127). All supporting data has been provided within the article or through supplementary data files. Two supplementary tables are available with the online version of this article.

**Impact Statement**

The genomic surveillance of SARS-CoV-2 has helped identify variants that are of concern to public health and accurately track the viruses’ spread. However, the dynamics of the virus transmission change as the pandemic progresses and population mobility normalizes. This work describes Omicron subvariant circulation in Mexico after the fourth wave and through the end of the fifth wave, in accordance with the fourth wave, which was dominated by the BA.1 Omicron subvariant, and contrary to reports from previous waves, the most abundant subvariants of BA.2, BA.4 and BA.5 exhibited nationwide distribution. However, minor subvariants had a localized circulation and were associated with introductions from a single geographical region; mainly, the southeast had a different dispersion dynamic, suggesting that it remains a point of entry for novel viruses to Mexico, and surveillance efforts will need to focus on this region.

## Introduction

Multiple variants of the severe acute respiratory syndrome coronavirus 2 virus (SARS-CoV-2) have been responsible for nearly 700 million cases and over 6.87 million deaths since December 2019 (Our World in Data, https://ourworldindata.org; accessed 6 March 2023). As of the time of writing, 2992 lineages have emerged globally according to the Phylogenetic Assignment of Named Global Outbreak (PANGO) classification (https://cov-lineages.org/lineage_list.html; accessed on 6 March 2023). The World Health Organization (WHO) has designated five of these SARS-CoV-2 viruses as Variants of Concern (VOCs) based on their increased epidemic potential, disease severity and immune escape, which contribute to faster spreading and replacement of prior variants, starting new epidemiological outbreaks, and requiring adjustments of public health protocols [1]. Alpha (B.1.1.7) was the first VOC to be detected [2] in December 2020, followed by Beta (December 2020) [3], Gamma (January 2021) [4], Delta (May 2021) [5] and Omicron (November 2021) [6]. Omicron (B.1.1.529 in the Pango nomenclature) was first reported in South Africa and Botswana in November 2021 [6]. It has 46 point mutations and three deletions (changes of two to three amino acids) compared to the Wuhan reference strain [7]; 30 of these changes are present on the spike protein alone, representing a major evolutionary leap compared with previous VOCs. In this work, a mutation is considered to belong to a variant if at least 90 % of the samples of the variant harbour it.

Immediately after its detection, Omicron was subclassified into variants BA.1, BA.2 and BA.3. These variants share 31 amino acid mutations and three deletions, in addition to their own set of mutations. In winter 2021, the BA.1 variant and its subvariants started a new epidemiological surge worldwide. Afterward, BA.2 superseded them as the predominant variant in the world [8, 9], allowing the epidemic wave of BA.1 to continue in some countries [10, 11], or causing a separate wave in other countries [12, 13], or leading to a small number of cases without an associated epidemic wave in others [14, 15]. The genomic sequences of BA.1 and BA.2 variants (and their subvariants) differ by 23 amino acids [6], resulting in enhanced replication capacity and transmissibility of BA.2 [9, 16, 17]. However, other reports indicated that people infected with this variant had a lower or equivalent death or hospitalization risk compared to BA.1 [18, 19] and a similar drop in the vaccine’s effectiveness, which was significantly lower for all Omicron variants [20].

Eventually, two additional variants, BA.4 and BA.5, emerged from the BA.2 clade [21] and quickly became dominant, driving a new epidemiological wave on a global scale. Compared to the BA.2 spike protein, the BA.4 and BA.5 sequences contain two additional amino acid changes (L452R and F486V), one deletion (69-70del) and one reversion (R493Q). These two variants were shown to have a higher infection rate in vaccinated or previously infected people [16, 22, 23] and a higher risk of hospitalization relative to BA.1 [10, 24].

Despite having a lower mortality rate than previous VOCs, the global spread of Omicron and its variants still poses a major challenge to public health. Genomic surveillance is therefore required to monitor the development and evolution of novel variants, which helps to assess the efficacy of diagnostic methods, the potential of immune escape, and the transmissibility of the virus. As previously described, the BA.1 variant (and its descending subvariants) caused Mexico’s fourth wave of infections [25]. On the other hand, BA.5 and, to a lesser extent, BA.4 were responsible for the fifth wave. In contrast, BA.2 dominated during the inter-wave interval and, in some States, at the start of the fifth wave, before BA.4 and BA.5 became dominant in the country. This study examines Mexico’s introduction, diversity, spread and distribution of these three variants.

## Methods

### Epidemiological data

Official records of daily cases and deaths were obtained from the open COVID-19 database from the General Epidemiological Directorate (Dirección General de Epidemiologia) (https://www.gob.mx/salud/documentos/datos-abiertos-152127) from 9 September 2022, update. The information was plotted according to the sample collection date using a 7-day moving average sliding window with a 1-day step. Epidemiological data processing and visualizations were created using the R programming language v. 4.2.2.

### Study design and ethical consideration

The samples and metadata used in this study are part of the national public health response to COVID-19, collected by the CoViGen-Mex consortium under the Official Mexican Standard NOM 017-SSA2-2012 (17) for the epidemiological surveillance programme. The samples were anonymous prior to the start of the study. In hospitals and clinics of the Mexican Social Security Institute (IMSS), oropharyngeal or nasopharyngeal swab samples were collected from all 32 states of Mexico. All IMSS hospitals are required to conduct an initial SARS-CoV-2 antigen test on 100 % of hospitalized patients and 10 % of outpatients. Then, 100 and 1 % of positive samples, respectively, undergo PCR testing. Between 1 April and 31 August 2022, 10 472 samples with a cycle threshold (Ct) value of 30 or less were collected. From these samples, if available, approximately 1400 were selected at random each month for sequencing, assuming the same percentage per state. A total of 7859(70.1 %) IMSS samples were processed for sequencing. Lastly, 59 samples from the Center for Research in Food and Development (CIAD) and 370 samples from the National Institute of Respiratory Diseases (INER) were included.

### High-throughput sequencing and subvariant identification

In total, 8288 SARS-CoV-2 samples were amplified with the Illumina COVIDSeq kit (Illumina, San Diego, CA, USA) using ARTIC 4.1 primers according to the manufacturer’s instructions (https://artic.network). The libraries were sequenced using Illumina NextSeq 550 or Miniseq platforms with 2×150 paired-end runs in differentinstitutions, forming the Consorcio de Vigilancia Genomica de Mexico (CoViGen-Mex). Basecalling was conducted with BCL Convert to generate FASTQ files. Adapters, low-quality bases, and duplicate reads were eliminated using FASTP v1.2 [26]. The remaining reads were mapped with Bowtie2 v2.3.4.3 [27] against the Wuhan-Hu-1 (NC_045512.1) reference genome to obtain consensus sequences using iVar (v1.3.1) [28], with the following parameters: base score *Q*>20, 20 minimum read coverage for basecalling, and a threshold under the majority rule (0.5 frequency) for single nucleotide polymorphisms (SNP). A total of 7022 sequences were uploaded to the EpiCoV database of the Global Initiative on Sharing All Influenza Data (GISAID) repository (https://www.gisaid.org/), as well as the GenBank database (Table S1)op[]\/i j4fvcsxz, bvfcwd with a mean genome coverage of 97.9 %, and a mean average sequencing depth of 2210.9 (±1001.5). For subvariant identification, the Pangolin software programme was utilized (https://github.com/cov-lineages/pangolin; accessed on 10 May 2022).

### SARS-CoV-2 Mexican sequences data set

A set of 9002 additional complete sequences from Mexico were downloaded from the GISAID database (accessed on 19 September 2022), covering the same period from 1 April to 30 August 2022. From this set, 23 Delta-classified genomes were discarded, resulting in 8979 additional genomes (Table S2). The 7022 sequenced genomes reported in this study and generated by the CoViGen-Mex consortium were added to this dataset, tallying 16 001 genomes from Mexico (Tables S1 and S2). The correlation between the frequency of variants in each State between these two datasets was 0.90(±0.5). The States of Aguascalientes, Coahuila, Nuevo León and San Luis Potosí were excluded from the correlation because in Aguascalientes, the consortium had sequenced fewer than ten genomes, and in the other four States fewer than 20 additional genomes were reported by other entities. Nayarit was excluded from all further analyses since it had only ten genomes. Notably, the sampling strategy carried out by other entities is distinct from that of the consortium; for example, some receive their samples from hospitals, some from private diagnostic laboratories, while others actively search for imported cases, among others. Despite the difference in sampling, the high correlation suggests that the genomic diversity observed during this period and described in the article has no sampling bias.

### Regional lineage distribution

To assess the differences in lineage distribution, a cluster analysis of the relative variant frequencies in each State was performed using R v.4.0.5 [29]. A State distances matrix was generated using the Vegan v.2.5.7 package [30]. Different parameters were evaluated: (a) dissimilarity distance measure; (b) method in agnes algorithm for assigning nodes to clusters, using the Cluster v.2.1.2 package [31]; (c) the number of optical clusters evaluated from two to eight groups. From the factoextra package [32], ‘silhouette widths’ were generated to evaluate the concept within-cluster cohesion and between-cluster isolation. Small positive or negative silhouette widths suggest poor State-cluster matches, while large positive widths indicate good fits. Results showed that Cao index was the best distance measure; the Ward method had the highest agglomerative coefficient (AC); and four clusters were the optimal, with an AC of 92 % and an average silhouette width of 0.49.

### Mutation, evolution and phylogenetic analyses

The genomes were analysed with NextClade CLI 1.9.0 (accessed on 20 November 2022) [33], with the default command-line parameters and relative to the Wuhan-Hu-1 reference genome to identify subvariants-specific mutations, insertions and deletions. For this purpose, NextClade was used to perform sequence alignment, variant calling and clade assignment on SARS-CoV-2 genomes while considering the geographical origin and mutational analysis of the genomes.

The covSPECTRUM logistic regression model was used, with the default parameters, to estimate the relative growth advantage per week of variants compared to co-circulating variants by using the estimated proportion of SARS-CoV-2 sequences with a specific mutation between 1 April and 31 August 2022, in Mexico [7] based on GISAID data. The provided relative growth reflects the advantage of variants spreading primarily through local transmission. In addition, an estimated logistic growth rate (per day) was estimated under the assumptions of intrinsic transmission advantage, immune evasion and a prolonged infectious period [34].

For each BA.2, BA.4 and BA.5 subvariant, an analysis to study their introductions to Mexico and their region of origin was carried out. To this end, the first 150 sequences of each variant detected in Mexico were selected, and geographical redundancy was eliminated to generate a set of 100 sequences that were used to identify closely related sequences with Audacity Instant available at http://www.gisaid.org. The resultant sequences were aligned using mafft v.7 [35] against the reference sequence and then processed with CD-HIT [36] to remove redundancy. A maximum-likelihood phylogenetic tree was inferred using iqtree2 [37] with the substitution model GTR+F+R3. The location of each sequence was set to the continent of origin, except for those from Mexico and the USA, given the large number of people who routinely cross the Mexico–USA border. An ancestral state reconstruction of the location was done using PastML [38], and the resulting trees were visualized in FigTree v1.4.4 (https://github.com/rambaut/figtree/issues/130).

To characterize the evolutionary spread of BA.4 and BA.5 variants circulating in Mexico, an individual subsample of sequences from their most abundant subvariants in the country was used. The sequences were selected to ensure they were representative of all states and months. Then, a subsample of the same subvariants from the rest of the world was included (Table S2). Finally, an alignment and a phylogenetic tree was done in the same way as described previously. The phylogenies were plotted using ggtree in R v.4.0.5 [29], colouring the tips by sampling location. Maps showing subvariants’ geographical distribution were constructed in R using the package mxmaps (https://github.com/diegovalle/mxmaps).

### Statistical analysis

All genomic information was expressed as counts and relative frequencies (percent). Using the median with interquartile data (IQR), the central tendency of these non-normally distributed data was described. Groups of states with a greater prevalence of a particular variant were identified as those with a higher interquartile relative frequency, as measured by month. The non-parametric Mann–Whitney U-test was utilized for group comparison. A *P*-value less than 0.05 was statistically significant (two-tailed). Statistical analyses were performed using the package ggplot in R v.4.0.5.

## Results

### Epidemiological information

**Fig. 1.**
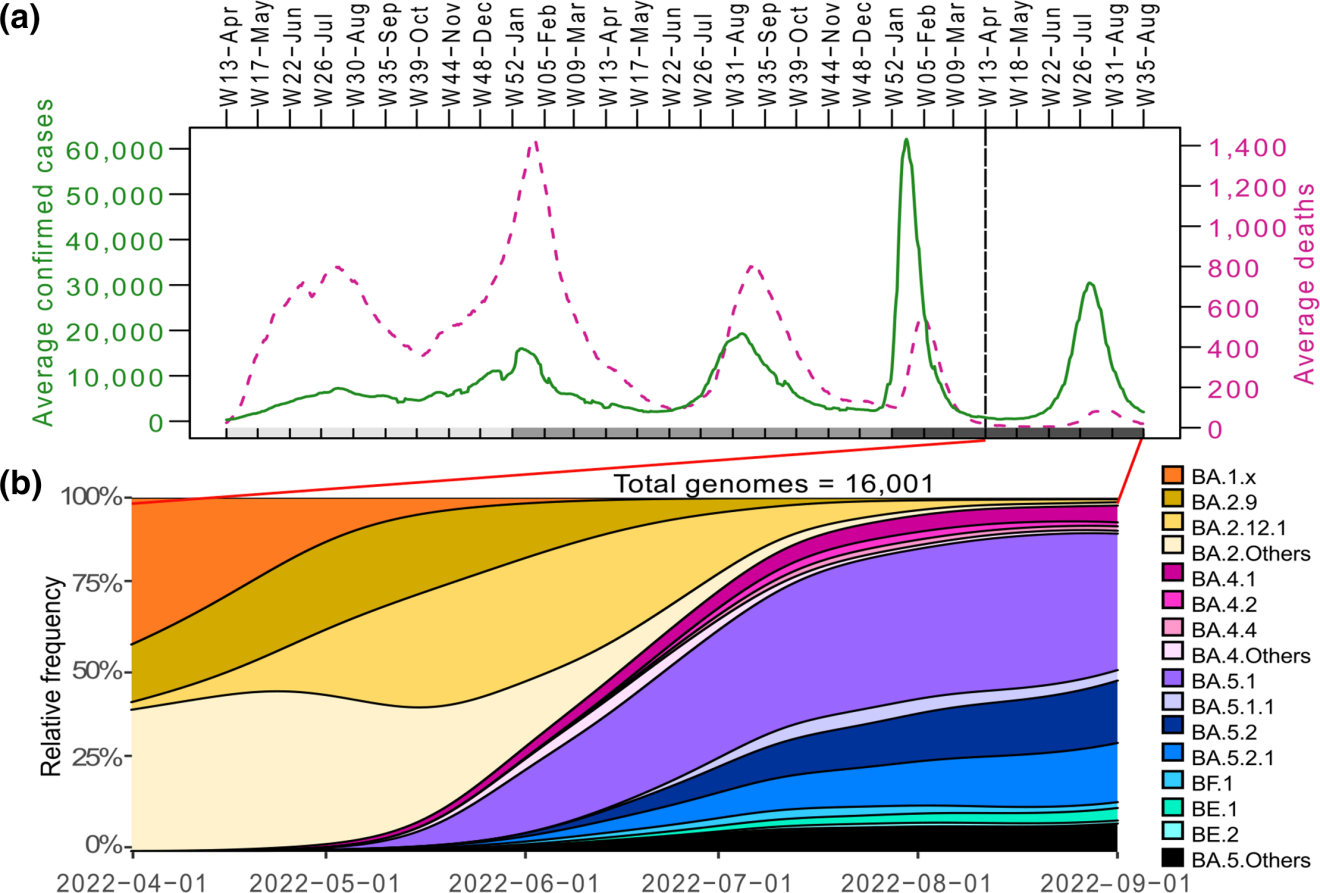
Epidemic data and SARS-CoV-2 subvariants circulating in Mexico, using a 7-day rolling average on a sliding window with a 1-day step. (**a**) Average daily COVID-19-positive cases and deaths in Mexico are shown in green and fuchsia, respectively. The years are denoted in the bars below (light grey, 2020; grey, 2021; dark grey, 2022). (**b**) Relative genome frequency of SARS-CoV-2 subvariants during the fifth epidemic surge and its preceding inter-wave period. Subvariants with <1 % of national abundance are collated as ‘Others’.

By September 2022, Mexico had traversed five epidemiological waves during the COVID-19 pandemic (Fig. 1a). Its fourth wave (Week [W] 51/December 2021 – W12/March 2022), driven by the BA.1 variant, reported the highest record of the 7-day moving average of daily cases, peaking at 62 134 on 15 January 2023, followed by the fifth wave (W22/Jun – W35/August 2022), which peaked at 30 475 cases on 10 July 2022. In contrast, the inter-wave period between the fourth and fifth waves (spanning W13/April – W21/May) was characterized by the lowest number of daily new confirmed cases in Mexico since the onset of the pandemic. Contrastingly, these two epidemic waves have recorded the lowest number of hospitalizations and deaths as of this date, with peaks of 555 (30 January 2022) and 87 (28 July 2022) daily deaths, respectively (7-day moving average), compared to 798 (17 July 2020), 1432 (22 January 2021) and 812 (12 August 2021) deaths in the preceding three waves. This decline in mortality was probably related to the increase in population immunity acquired either through vaccination or previous infections or a possible decrease in virulence of the circulating variants (Fig. 1a).

### Virus genetic diversity and geographical distribution in the fifth wave

The circulation of the SARS-CoV-2 variants in Mexico during the first 2 years of the pandemic has been described previously [25, 39–41]. Fig. 1b depicts the relative frequency of subvariants in Mexico between April and August 2022, which includes the last inter-wave period and the fifth wave. During this time, the BA.2, BA.4 and BA.5 variants (and related subvariants) dominated the infections in the country. A total of 89 distinct SARS-CoV-2 Omicron subvariants were identified (Table S1), but only a few circulated throughout the country.

The emergence of the BA.2 variant occurred at the beginning of the inter-wave period. In some other countries, BA.2 caused a second Omicron wave different from the BA.1 wave [12, 13]. In Mexico, BA.2 spread predominantly between waves (March to May 2022) and was displaced at the beginning of the fifth wave by BA.4 and BA.5. Forty-six different subvariants of BA.2 were identified in Mexico, with a cumulative relative frequency of 24 % in April, 90 % in May, and later descending to 52 % in June (Fig. 1a). The two most widespread BA.2 subvariants were BA.2.9 and BA.2.12.1 (Table 1 and Fig. 1b).

**Table 1.** Monthly frequency (%) of Omicron subvariants in Mexico between April and August 2022

Variant	Subvariant	No. of genome sequences (frequency in %)				
		April / 22	May / 22	June / 22	July / 22	August / 22
BA.1	BA.1	271(34.3 %)	82(4.0 %)	21(0.4 %)	4(0.1 %)	6(0.2 %)
BA.2	BA.2.9	150(19.0 %)	463(22.6 %)	447(9.0 %)	55(1.1 %)	9(0.3 %)
	BA.2.12.1	39(4.9 %)	679(33.1 %)	1590(32.0 %)	333(6.9 %)	44(1.3 %)
	BA.2.Others	320(40.6 %)	707(34.5 %)	555(11.2 %)	111(2.3 %)	39(1.2 %)
BA.4	BA.4.1	1(0.1 %)	20(1.0 %)	226(4.5 %)	240(5.0 %)	146(4.3 %)
	BA.4.2	0(0.0 %)	0(0.0 %)	40(0.8 %)	129(2.7 %)	52(1.5 %)
	BA.4.4	0(0.0 %)	0(0.0 %)	27(0.5 %)	94(1.9 %)	39(1.2 %)
	BA.4.Others	0(0.0 %)	19(0.9 %)	215(4.3 %)	50(1.0 %)	36(1.1 %)
BA.5	BA.5.1	1(0.1 %)	50(2.4 %)	1169(23.5 %)	1914(39.5 %)	1397(41.4 %)
	BA.5.1.1	0(0.0 %)	0(0.0 %)	23(0.5 %)	230(4.7 %)	108(3.2 %)
	BA.5.2	0(0.0 %)	1(0.0 %)	180(3.6 %)	526(10.9 %)	547(16.2 %)
	BA.5.2.1	0(0.0 %)	5(0.2 %)	194(3.9 %)	505(10.4 %)	510(15.1 %)
	BF.1	0(0.0 %)	1(0.0 %)	64(1.3 %)	147(3.0 %)	54(1.6 %)
	BE.1	0(0.0 %)	3(0.1 %)	43(0.9 %)	107(2.2 %)	110(3.3 %)
	BE.2	0(0.0 %)	0(0.0 %)	5(0.1 %)	47(1.0 %)	32(0.9 %)
	BA.5.Others	0(0.0 %)	11(0.5 %)	165(3.3 %)	351(7.2 %)	242(7.2 %)

**Fig. 2.**
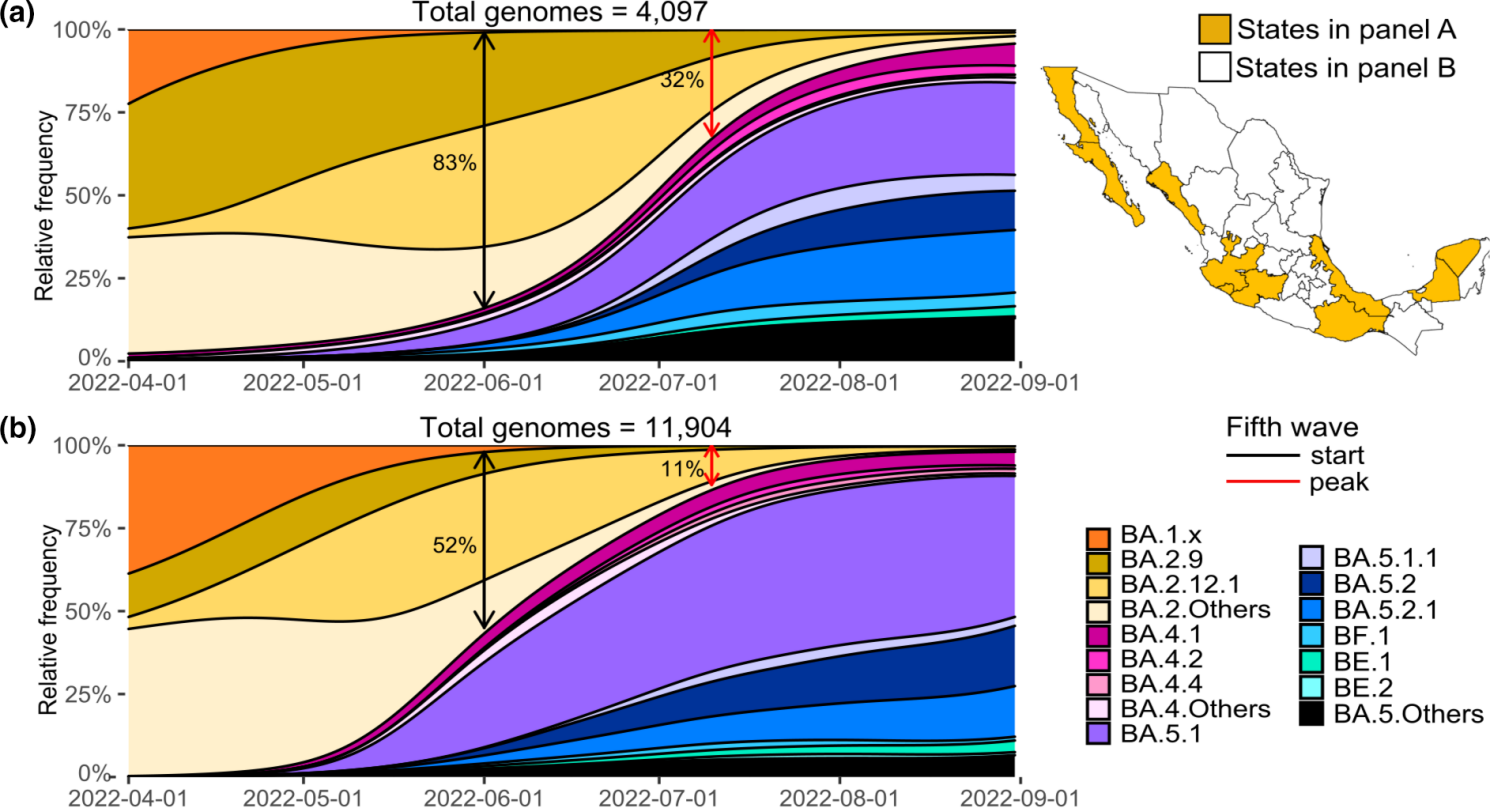
Comparison of SARS-CoV-2 genomic subvariant diversity in States with high and low BA.2 circulation in Mexico during the fifth wave of infections. (**a**) States where BA.2 was more abundant at the beginning (black arrow) and peak (red arrow) of the wave. The map on the right highlights those States. (**b**) Remaining States, where BA.2 was not as abundant during the start of the wave and was early displaced by BA.5 (States shown in white on the map).

In some States, BA.2 subvariants, such as BA.2.12.1 and BA.2.9, drove the rise of the fifth wave (Fig. 2), showing a cumulative relative frequency higher than the interquartile (>67 %) in June, the beginning of the wave. These States are located either in the West, on the Pacific coast (Baja California, Baja California Sur, Colima, Jalisco, Michoacan, Oaxaca and Sinaloa) or in the East, in the Gulf of Mexico (Campeche, Veracruz and Yucatan). The median abundance in June was significantly higher in these States than in the rest median=71 %(95 % CI: 65 %,72 %), vs. median=45 %(95 % CI: 41–55 %), Mann–Whitney U test *P*<0.00001. Fig. 2a depicts SARS-CoV-2 subvariant circulation from April to August 2022 in these States with a higher abundance of BA.2(10 out of 32 States; see the map to the right of the figure for their location). The BA.2 variant reached a cumulative relative frequency of 83.1 % in June and 32.2 % during the epidemiological peak of July. On the other hand, (Fig. 2b) depicts the rest of the country (States with a lower BA.2 abundance), where BA.2 reached 52.2 and 11.2 % at the beginning and peak of the wave, respectively. In addition, (Fig. 2a) shows that BA.2.9 circulated during the late inter-wave period, while BA.2.12.1 also extended further into the fifth wave.

In May 2022, BA.5 began to expand, overtaking BA.2 by late June as the most abundant variant (Fig. 1b). This replacement coincided with the acceleration of the fifth epidemiological wave (Fig. 1a), with BA.5.1 being the most frequent subvariant. BA.4 emerged around the same time as BA.5, but its national relative frequency remained below 10 % throughout the wave, with BA.4.1 being the most common of its subvariants. In contrast, the BA.5 subvariants reached over 90 % by the end of August 2022 (Table 1). This observed increase is in line with the growth advantage analysis results, which indicated that BA.4 and BA.5 variants have a higher daily growth rate than BA.2, at 0.06(95 % CI: 0.06–0.07) and 0.09(95 % CI: 0.09–0.10), respectively [7]. These values correspond to an estimated relative weekly growth advantage of 57 %(95 % CI: 53–60 %) and 90 %(95 % CI: 86–93 %) for BA.4 and BA.5 over BA.2, respectively [34].

### Analysis of the introduction of BA.2, BA.4 and BA.5 subvariants to Mexico

During the fifth wave, no distinct geographic regions (groups of contiguous States) of SARS-CoV-2 virus subvariant circulation were observed (Fig. S1), as was the case during the fourth wave with the variant BA.1 in Mexico [25]. Even though four groups were formed, one of them, in blue, contains most of the States. Another cluster, in red, consists of dispersed states located from north to south. Moreover, the minor or negative values of the silhouette width measure in several states suggested that these states were incorrectly grouped. Interestingly, the lack of geographical clustering was observed for the most abundant subvariants, such as BA.2.12,1, BA.4.1, BA.5.1, BA.5.2 and BA.5.2.1 (Table 1). However, other subvariants with a lesser abundance, such as BA.2.9, BA.4.2, BA.4.4 and BA.5.1.1, showed a distinctive circulation in non-neighbouring states. It is not easy to assess the reasons for the differences in variant spread because numerous stochastic events are possible. One area worth exploring was analysing the relative significance of importation events from different geographical areas versus virus spread within Mexico. To determine the origins of all Mexican major subvariants (clades composed by a minimum of two sequences from Mexico directly descending from another sequence of Mexico), the ancestral states of sequence location were reconstructed as described in Methods. Table 2 lists the number of introductions that resulted in Mexican clades and their most likely origin.

**Table 2.** Clades of Mexican sequences (local transmission), along with the percentage of their ancestral nodes in each locality

Variant	Subvariant	No. clades	USA	Europe	North america	South America	Asia	Ambiguous
BA.2	BA.2.9	10	5(50.0 %)	1(10.0 %)	0(0.0 %)	2(20.0 %)	1(10.0 %)	1(10.0 %)
	BA.2.12.1	20	19(95.0 %)	0(0.0 %)	0(0.0 %)	0(0.0 %)	0(0.0 %)	1(5.0 %)
BA.4	BA.4.1	11	3(27.3 %)	4(36.4 %)	0(0.0 %)	1(9.1 %)	0(0.0 %)	3(27.3 %)
	BA.4.2	12	11(92.0 %)	0(0.0 %)	0(0.0 %)	0(0.0 %)	0(0.0 %)	1(8.0 %)
	BA.4.4	15	9(60.0 %)	0(0.0 %)	0(0.0 %)	1(6.7 %)	0(0.0 %)	5(33.3 %)
BA.5	BA.5.1	10	2(20.0 %)	3(30.0 %)	0(0.0 %)	2(20.0 %)	1(10.0 %)	2(20.0 %)
	BA.5.1.1	16	14(88.0 %)	0(0.0 %)	0(0.0 %)	0(0.0 %)	0(0.0 %)	2(12.0 %)
	BA.5.2	17	1(0.06 %)	6(35.0 %)	0(0.0 %)	0(0.0 %)	3(18.0 %)	7(41.0 %)
	BA.5.2.1	12	4(33.0 %)	3(25.0 %)	0(0.0 %)	2(17.0 %)	1(8.0 %)	2(17.0 %)
	BF.1	10	8(80.0 %)	1(10.0 %)	0(0.0 %)	0(0.0 %)	0(0.0 %)	1(10.0 %)
	BE.1	11	2(18.2 %)	4(36.4 %)	1(9.1 %)	0(0.0 %)	0(0.0 %)	4(36.4 %)
	BE.2	11	11(100 %)	0(0.0 %)	0(0.0 %)	0(0.0 %)	0(0.0 %)	0(0.0 %)

Some subvariants frequently had their origins in the USA, in agreement with the regular number of people crossing the Mexico–USA border and the influx of tourists from the USA. Other subvariants had their origins in Europe and may have also been imported through tourism, followed by others whose ancestral origins were in South America and Asia. Of note, the analysis could not discern the origin of certain Mexican clades, particularly for BA.4.1, BA.4.4, BA.5.2 and BE.1.

In the case of BA.2 subvariants, BA.2.9 was introduced at least ten times, resulting in Mexican clades that indicate further spread within Mexico (Fig. S2). Half of these introductions originated from the USA, and the rest came from other continents (Fig. S2 and Table 2). In contrast, BA.2.12.1 was mainly imported from the USA (Table 2). As shown in Fig. S3, this subvariant was widespread in the USA, and most sequences sampled with Audacity Instant were from this country.

Regarding BA.4, the most widespread subvariant in the country, BA.4.1, was introduced to Mexico from different continents, mainly from Europe (see Fig. S4). In contrast, BA.4.2 and BA.4.4 were mainly imported from the USA, and in both instances, the largest Mexican sequence clades originated in the USA (Figs S5 and S6).

The origin of the introduction of BA.5 subvariants as BA.5.1 was highly variable, reflecting the worldwide circulation of this subvariant, as indicated by the geographical diversity of the sequences sampled with Audacity Instant (Table 2 and Fig. S7). BA.5.1.1 was predominantly introduced from the USA, despite the detection of most of its sequences in southern Mexico (Fig. S8 and below). On the other hand, the origins of many BA.5.2 introductions could not be determined, but 35 % were from Europe (Fig. S9). The origin of BA.5.2.1 was also diverse, without a single dominant region, as shown in Fig. S10.

BF.1 is particularly interesting since it shows a large clade of Mexican sequences whose geographical origin cannot be established (Fig. S11). Other Mexican clades, predominantly of USA origin, were found despite their detection primarily in the south of Mexico (see Characterization of the BA.4 and BA.5 variants in Mexico below). In contrast, Mexican BE.1 clades originated mainly in Europe (Fig. S12) and circulated primarily in central Mexico. Finally, BE.2 viruses were introduced from the USA, where most subsampled sequences originated (Fig. S13).

### Characterization of the BA.4 and BA.5 variants in Mexico

**Fig. 3.**
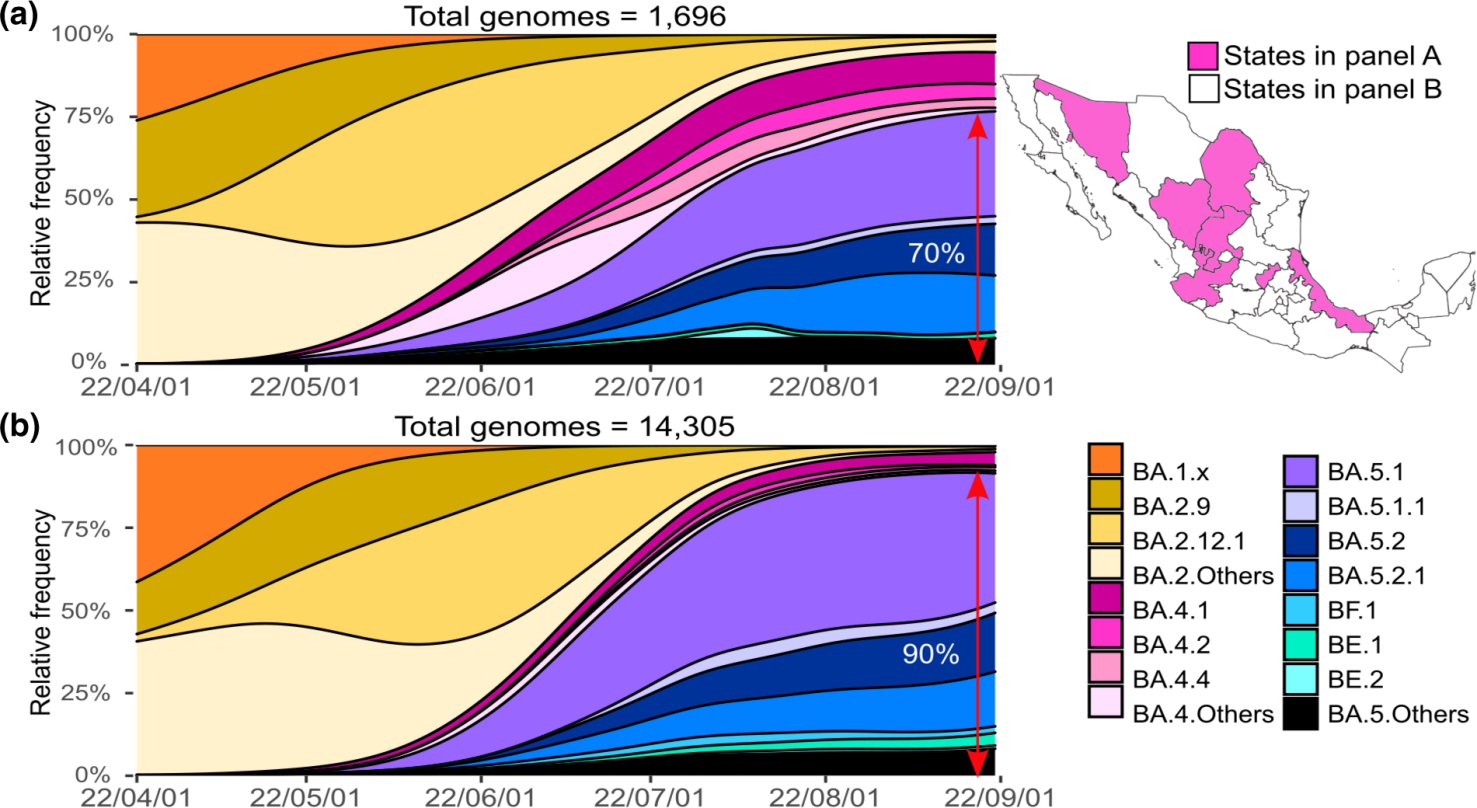
Comparison of SARS-CoV-2 genomic subvariant diversity in States with high and low BA.4 circulation in Mexico during the fifth wave of infections. (**a**) Sequenced subvariants in the States where BA.4 relative frequency was >20 %, which are shown in fuchsia in the map on the right. (**b**) Sequenced subvariants in the remaining States, with a lower abundance of BA.4, which are shown in white on the map.

In April 2022, the BA.4 variant and its subvariants were identified in Mexico with a low national relative frequency (less than 1 %) and a maximal abundance in June and July (around 10 %). However, in Coahuila, Durango, Jalisco, Queretaro, Sonora, Veracruz and Zacatecas, the cumulative relative frequency of BA.4 exceeded the interquartile abundance value of 20 % in June and 17 % in July. The median abundance of BA.4 in these States was significantly higher than in the rest of the country median=30 %(95 % CI: 31 %,38 % vs. median=10 %(95 % CI: 9–13 %), Mann–Whitney U test *P*<0.00001. In these States, BA.5 reached a maximum relative frequency of 70 % in late August (Fig. 3a). In contrast, in States with low BA.4 circulation, BA.5 accounted for 90 % of all identified genomes in the same period (Fig. 3b). The difference is probably due to the early introduction of BA.4 in these states, and the minor advantage of BA.5 over BA.4, which is only a daily growth rate of 0.02(95 % CI: 0.02–0.02 %). In states with limited circulation of BA.4, BA.5 competed with BA.2, and was able to displace it faster. Likewise, in these states with higher BA.4 abundance, at least three of its subvariants circulated at different points (Fig. 3a), whereas in the remaining states only one variant circulated.

**Fig. 4.**
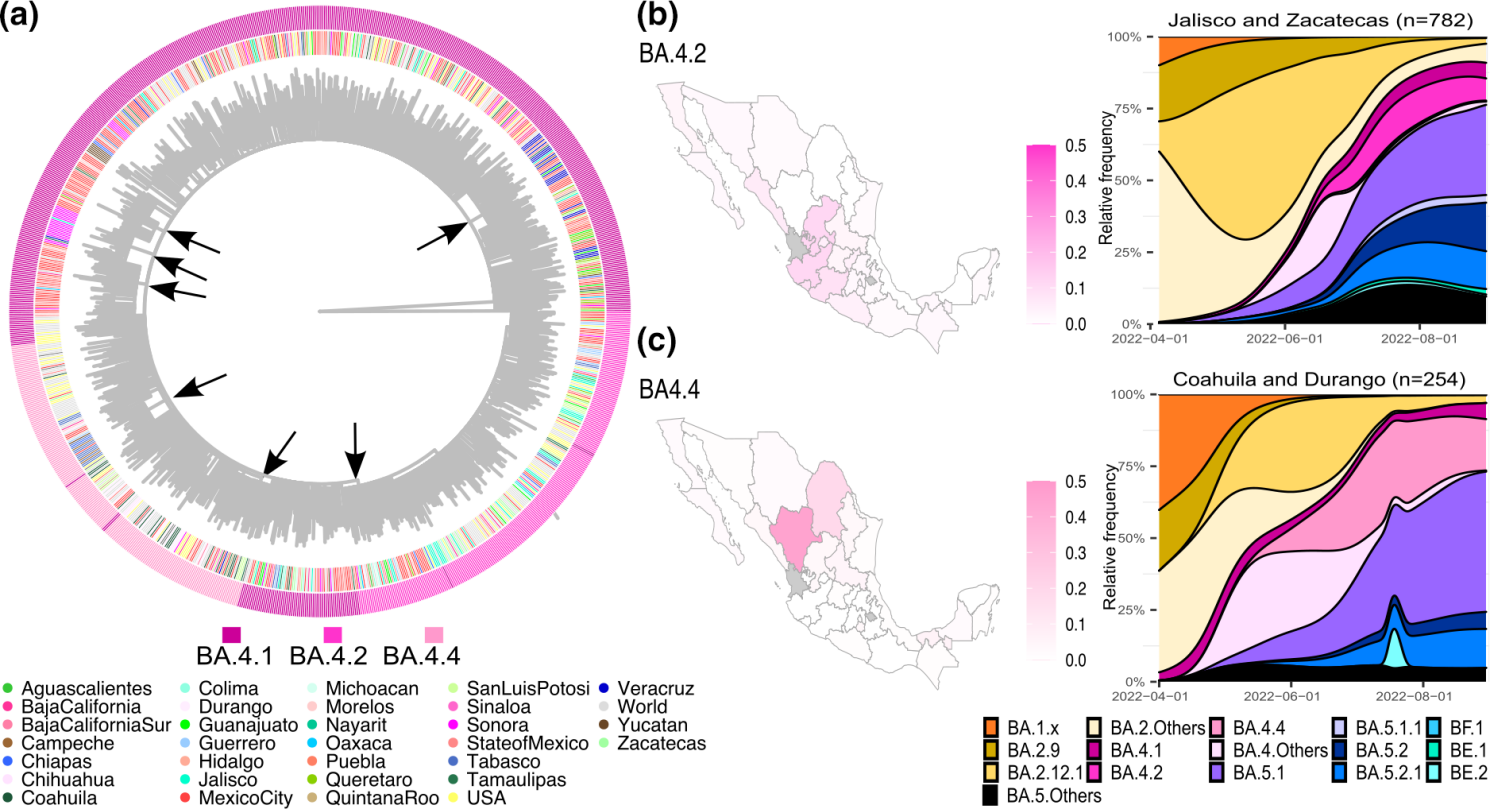
BA.4 variant in Mexico during fifth wave. (**a**) Time-scaled maximum-likelihood phylogenetic reconstruction of the three most abundant BA.4 subvariants in Mexico (BA.4.1, BA.4.2 and BA.4.4). The place of origin is shown in the inner circle, while the subvariants are displayed in the outer circle. (**b**) Geographical distribution of BA.4.2 and BA.4.4. (**c**) Longitudinal abundance of subvariants where BA.4.2 and BA.4.4 were abundant. States without BA.4 sequences are shown in grey.

Six subvariants of BA.4 circulated in the country (Table S1), BA.4.1 being the most common, followed by BA.4.2 and BA.4.4. To determine how the BA.4 variant spread, a phylogenetic reconstruction was carried out (Fig. 4a), using all the sequences from Mexico of these three subvariants and a set of sequences from other countries obtained during the same period. The phylogeny shows a few clusters of Mexican sequences from a single or a few States, suggesting local transmission. These groups are marked with arrows in (Fig. 4a). However, these events were not common enough to overcome a country-wide dispersion dynamic for BA.4.1, which was the most abundant subvariant. In contrast, BA.4.2 and BA.4.4 had more restricted geographical distributions (Fig. 4b, c). The relative frequency of BA.4.2 reached a maximum of 15 % in the states of Jalisco and Zacatecas (Fig. 4b), while BA.4.4 exceeded 22 % in Durango and Coahuila (Fig. 4c). In the rest of the country, the frequency of these two subvariants remained lower than 6 %.

**Fig. 5.**
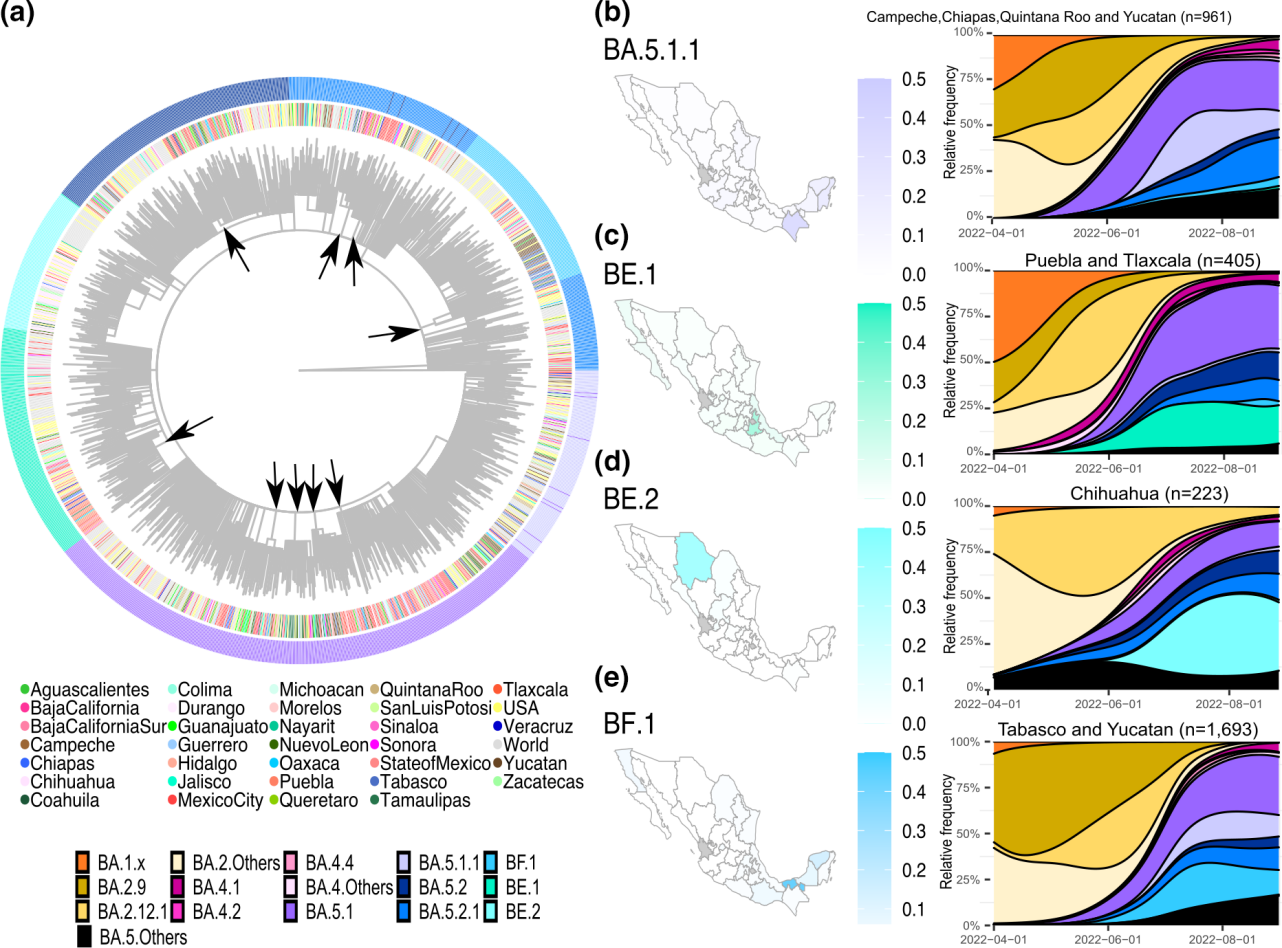
BA.5 variant in Mexico during fifth wave. (**a**) Time-scaled maximum-likelihood phylogenetic reconstruction of the seven most abundant BA.5 subvariants (BA.5.1, BA.5.1.1, BA.5.2, BA.5.2.1, BE.1 (alias of B.1.1.529.5.3.1.1), BE.2 (alias of B.1.1.529.5.3.1.2) and BF.1 (alias of B.1.1.529.5.2.1.1). The place of origin is shown in the inner circle, while the subvariants are displayed in the outer circle. Panels (b) through (e) reflect the geographical distribution and longitudinal abundance of a specific subvariant. (**b**) BA.5.1.1. (**c**) BE.1. (**d**) BE.2. (**e**) BF.1. States without sequences are in grey.

In Mexico, the BA.5 variant was first identified in April 2022. During the fifth wave, 24 subvariants were identified, summing a relative frequency of 0.1 % in April, 3.5 % in May, 37.1 % in June, 79.0 % in July, and 90.0 % in August. By August 2022, its four most abundant subvariants were BA.5.1, BA.5.1.1, BA.5.2 and BA.5.2.1, accounting for 72.7 % of the July reported sequences (Table 1). A phylogenetic reconstruction, abundance maps and stacked density plots were utilized to characterize the longitudinal and regional distribution of BA.5 in Mexico (Fig. 5). Like BA.4, the phylogeny suggests multiple local transmission events, with clades of only Mexican sequences (arrows in Fig. 5a).

Moreover, BA.5.1, BA.5.2 and BA.5.2.1 subvariants were widespread across the country (Fig. 5a), but other subvariants had a more restricted circulation. For example, BA.5.1.1 circulated predominantly in the southeast, particularly in the States of Chiapas and Quintana Roo, with a maximum relative frequency exceeding 30 % (Fig. 5b), followed by Yucatan (>18 %) and Campeche (>9 %). Furthermore, BE.1 (alias of B.1.1.529.5.3.1.1) circulated mainly in Puebla and Tlaxcala (central Mexico; Fig. 5c), while BE.2 (B.1.1.529.5.3.1.2) in Chihuahua (northern Mexico; Fig. 5d), with peaks reaching more than 18 and 37 %, respectively. On the other hand, BF.1 (B.1.1.529.5.2.1.1) was frequent in the South (Fig. 5c), especially in Tabasco (with >50 %) and Yucatan (>15 %) (Fig. 5e).

### Comparative mutation profiles of BA.2, BA.4 and BA.5 subvariants

**Fig. 6.**
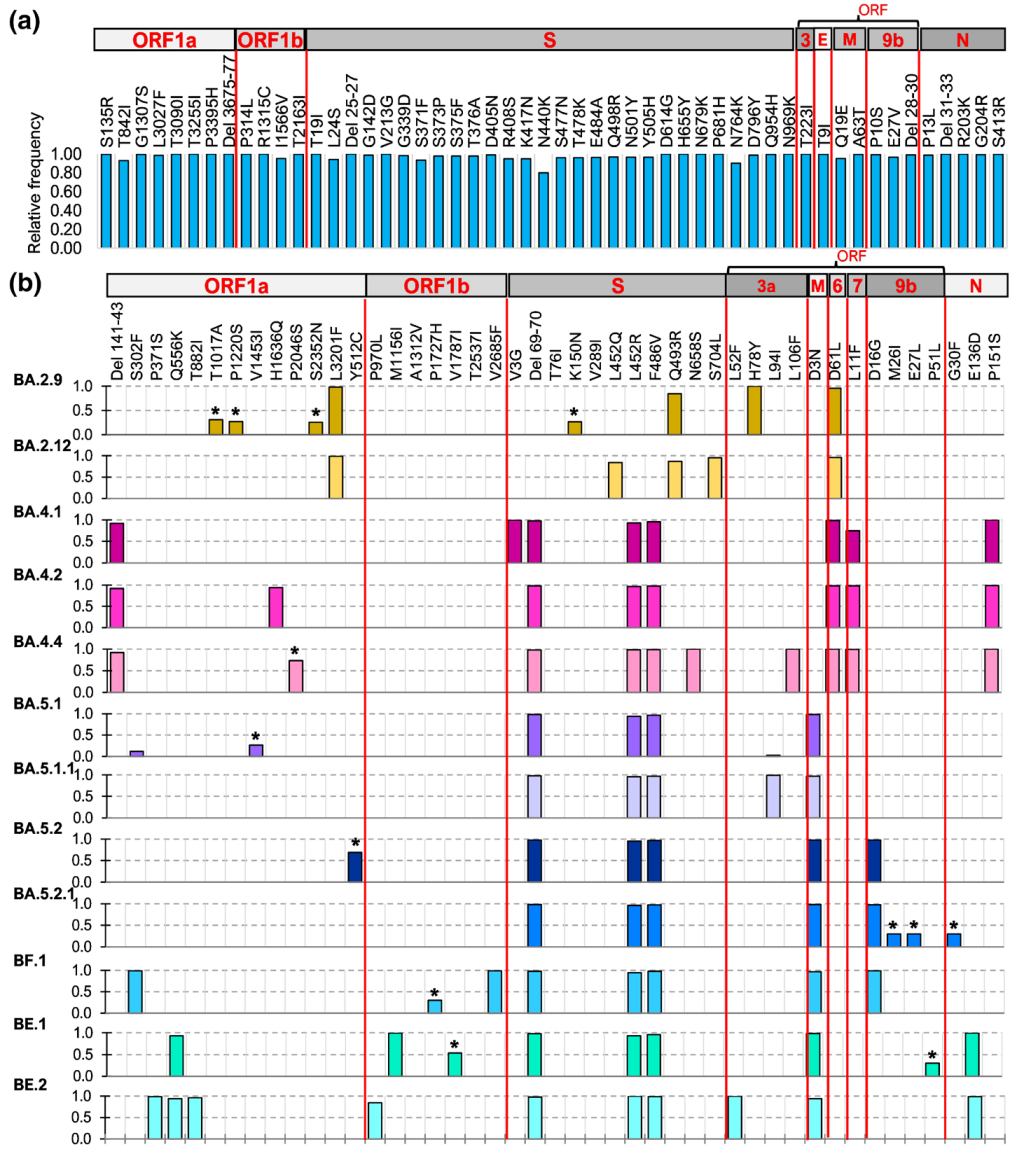
Mutation profiles of BA.2, BA.4 and BA.5 subvariants in Mexico. (**a**) Shared mutations in all three variants. (**b**) Proportions of defining mutations in each subvariant (above 0.8) and other minor mutations identified only in Mexico denotated with the sign *.

In the 16 001 Mexican SARS-CoV-2 genomes reported between April and August 2022, 12 119 different nucleotide and 6835 amino acid mutations were identified relative to the Wuhan-Hu-1 reference genome, but only 189 and 121, respectively, had a relative frequency above 1 %. Only amino acid substitutions and deletions with an abundance greater than 25 % for each subvariant were analysed, however the complete list of mutations can be found in Table S3. All genomes from variants BA.2, BA.4 and BA.5 shared 48 amino acid mutations and four deletions of three amino acids each (frequency proportion >0.8), with 27 mutations and one deletion occurring in the spike (S) protein. The relative frequency of these canonical mutations in the Mexican genomes is shown in (Fig. 6a). This observation is consistent with the reference genomes of these variants [7].

BA.2 variant had two additional specific amino acid changes (ORF1a:L3201F and S:Q493R), while BA.4 had three (ORF1a:Del141/143, ORF7b:L11F and N:P151S), and BA.5 had one (M:D3N) (Fig. 6b). BA.2 and BA.4 shared one mutation (ORF5:D61L), whereas BA.4 and BA.5 shared three (S:Del69/70, S:L452R, and S:F496V). In addition, the genomes of BA.2.9, BA.2.12, BA.4.1, BA.4.2, BA.4.4, BA.5.1, BA.5.1.1, BA.5.2, BA.5.2.1, BF.1, BE.1 and BE.2 subvariants contain between one and four additional specific mutations (Fig. 6b). Interestingly, a few additional substitutions with a lower frequency proportion (<0.4 %) were identified in BA.2.9, BA.5.1, BA.5.2.1, BF.1 and BE.1 (* in Fig. 6b), which may have been acquired in Mexico as they were absent from sequences from other countries.

## Discussion

The fifth wave of the COVID-19 pandemic in Mexico (June–August 2022) had the second-largest average number of new cases per day, just below the fourth wave (December 2021–March 2022); however, it had the lowest numbers of hospitalizations and deaths. On the other hand, the period between the fourth and fifth waves had the fewest daily cases reported since the start of the pandemic. During this inter-wave time, BA.2 dominated the variant landscape, with BA.2.9 and BA.2.12.1 being the predominant subvariants in the country. The first one was introduced multiple times from different parts of the world, while the latter one was most frequently introduced from the USA, where it reached a relative frequency of 62 % [7]. Like in other countries [14], in Mexico, BA.2 spread was not associated with increased reported cases and circulated predominantly in the inter-wave period. This variant was later superseded by BA.4 and BA.5, which are closely related to BA.2 and share many mutations. Although the reason for the lack of an epidemic peak associated with BA.2 is unknown, probably its introduction to the country shortly after the fourth wave, which resulted in a very high number of infections, together with the application of booster vaccine shots to a large part of the population in the previous months, were contributing factors [25]. Interestingly, BA.2 and its subvariants continued to circulate halfway into the fifth wave in the States of the Pacific coast (Baja California, Baja California Sur, Colima, Jalisco, Michoacan, Oaxaca and Sinaloa) and Gulf of Mexico (Campeche, Veracruz and Yucatan) but were eventually displaced by BA.5 subvariants, probably due to the growth advantage of the latter.

While the first three waves of the pandemic in Mexico showed strong geographical clustering of lineage circulation (specific variants were strongly associated with specific regions) [39, 40], in this study, it was found that the most abundant subvariants in the inter-wave period (BA.2.12.1 and BA.2.9) and fifth wave (BA.4.1, BA.5.1 and BA.5.2) did not exhibit geographical clustering. The reasons for these viral dispersion disparities are complex. For instance, lifting restrictions and sanitary measures in Mexico and around the world may increase mobility, resulting in more introductions of the same variant into the country from different regions of the world and more clusters of local transmission. This may speed the spread of viruses with nearly the same genomic sequence in different parts of the country. This contrasts with a scenario of worldwide mobility restrictions with fewer successful introductions, resulting in viruses spreading mostly through continuous chains of transmission within Mexico as well as locally acquired mutations that cause geographical clustering. For instance, it has been reported that seven introductions resulted in transmission chains for the Alpha variant, whereas Gamma and Delta had two and six, respectively [42]. Although the methodology employed in the present study differs and is focused on each subvariant’s early introduction events, this work identified 10 to 17 Mexican clades in these early trees. Further analysis will be needed to assess how other factors, such as previous immunity and population mobility, affect virus spread as the pandemic progresses, but here we provide data that suggests the number of successful introductions that result in local transmissions play a role in viral dispersion.

On the other hand, some less frequently observed subvariants, such as BA.4.2, BA.4.4, BA.5.1.1, BE.1, BE.2 and BF.1, were more abundant in specific Mexican States for at least 1 month, suggesting restricted local circulation. This is supported by the identification of Mexican subclades in the phylogenies, and their origins were more from one specific region. The States in which these subvariants circulated varied; in the case of BA.4.2 and BA.4.4 was in contiguous States in Mexico’s central regions, whereas BA.5.1.1 BE.1, BE.2 and BF.1 in the southeast or single isolated States. The results of this study support previous ones [25, 39, 40], showing that the southeast region may be a major entry point and contributed to the introduction and evolution of novel variants in Mexico [43].

Interestingly, BA.5 did not exceed 70 % in States where BA.4 circulation was significant, regardless of subvariant, whereas it accounted for over 90 % in those that did not. This observation is most likely due to the early entry of BA.4 in these States and BA.5’s marginal growth rate advantage of 0.02 over BA.4 [7], which was insufficient to overcome BA.4 during the fifth wave. However, compared to BA.2, BA.5 showed a relative growth rate advantage of 0.1 per day [7], allowing it to outgrow this variant. The difference lies in the specific mutations that give BA.5 and BA.4 an edge over the BA.2 variant. The presence of L452R and F486V mutations in the spike (S) protein, which are defined changes in the BA.4 and BA5 variants, has been linked to their ability to outcompete other variants; mutation S:L452R has independently evolved within the Delta and Omicron viruses under convergent evolution, and an alternative related mutation (S:L452Q) is instead present in BA.2.12.1. The transmission advantage of these viruses has been suggested to be attributable to these two spike mutations [44, 45]. In addition, these mutations may increase the receptor affinity of BA.4 and BA.5 subvariants [46], which most probably contributes to their rapid spread. Despite having identical mutations in their spike proteins, BA.5 spread more successfully than BA.4 in Mexico and worldwide. Although most differential mutations remain uncharacterized, mutation ORF6:D61L has been described as an interferon (IFN) antagonist that inhibits mRNA export via its interaction with the nuclear pore complex. This mutation compromises the function of ORF6, thereby preventing the virus’s innate immune escape, which has been suggested to contribute to the difference in the expansion rate of BA.4 and BA.5 [47].

The emergence of novel subvariants of the SARS-CoV-2 virus, such as Omicron, demonstrates the virus’s rapid rate of mutation and emphasizes the necessity for continuous surveillance. These subvariants were found to be more transmissible, infectious, and have a higher immune evasion. Regular and timely sequencing of the virus at local scales and worldwide is still essential to monitor the evolution of SARS-CoV-2 virus in real-time and to determine mitigation strategies to minimize infections and reduce healthcare demand in Mexico. Moreover, understanding the genetic makeup of the virus and its subvariants can provide critical insights into potential vaccine targets and inform the development of next-generation vaccines and treatments.

## Supplementary Data

Supplementary material 1

Supplementary material 2
