# Supplementary material for: SARS-CoV-2 Omicron variants BA.4 and BA.5 dominated the fifth COVID-19 epidemiological wave in Mexico
Source: Microb Genom. 2023 Dec 19;9(12):001120. doi: 10.1099/mgen.0.001120 (PMC10763511; doi:10.1099/mgen.0.001120)

Fig. S1. Cluster analysis of the relative frequencies of subvariants among the States of Mexico. A) Map of Mexico with the four geographical regions indicated B) Cluster dendrogram with the four groups. C) Comparative average Silhuoutte widths of the clusters, evaluating within-cluster cohesion and between-cluster isolation.

A)

B)

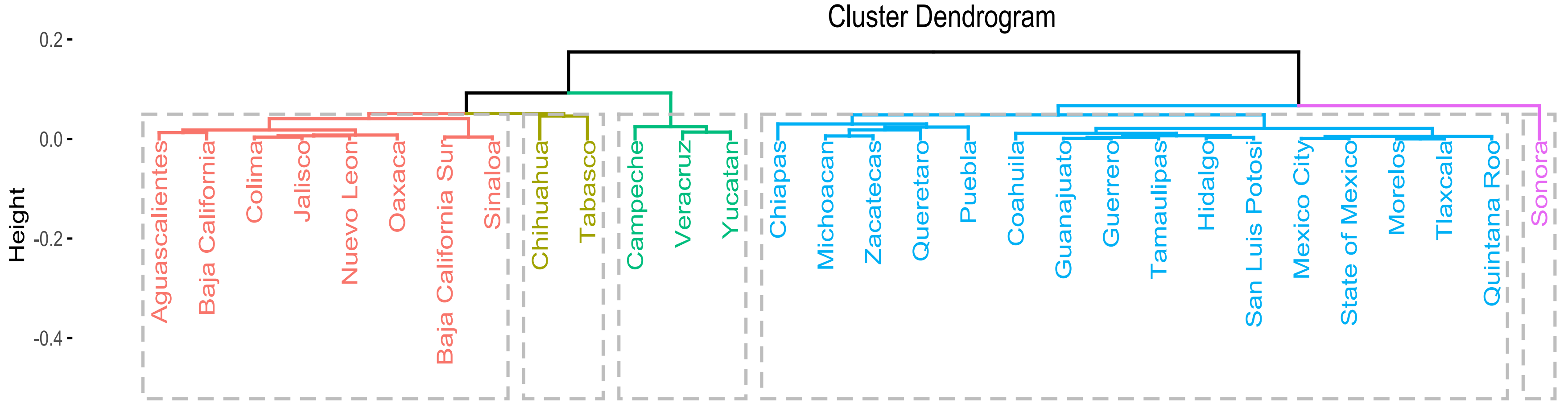

C)

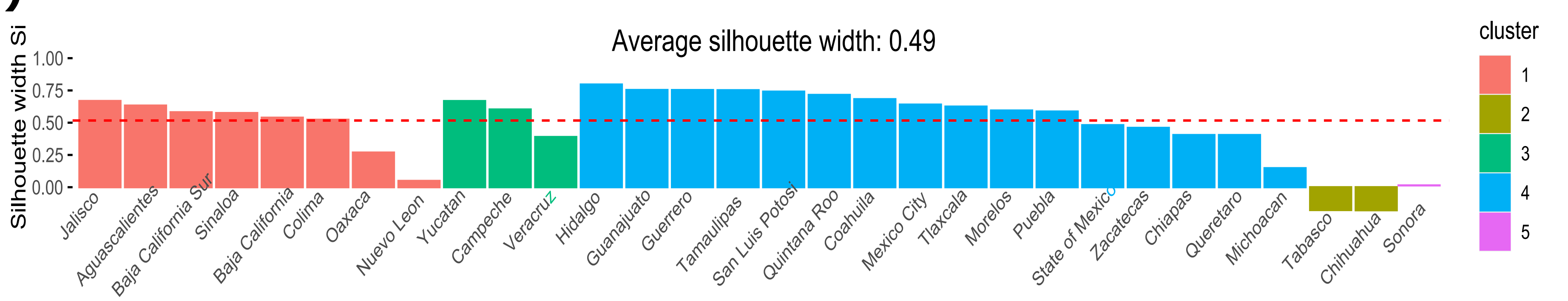

Fig. S2. BA.2.9

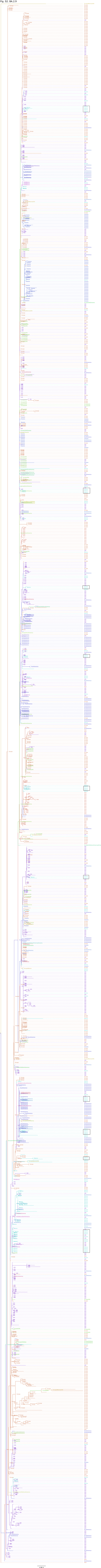

Fig. S3. BA.2.12.1

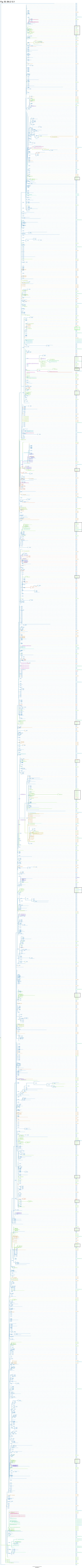

Fig. S4. BA.4.1

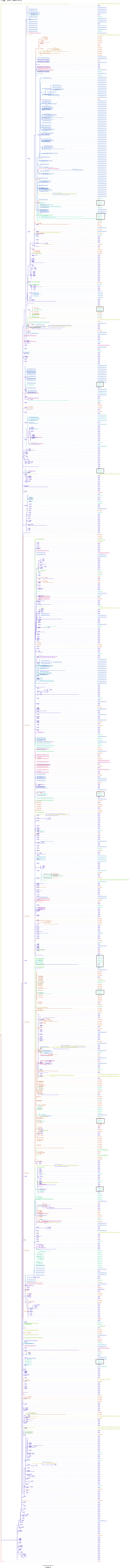

Fig. S5. BA.4.2

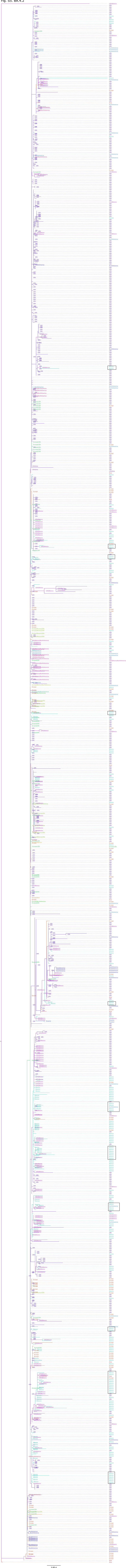

Fig. S6. BA.4.4

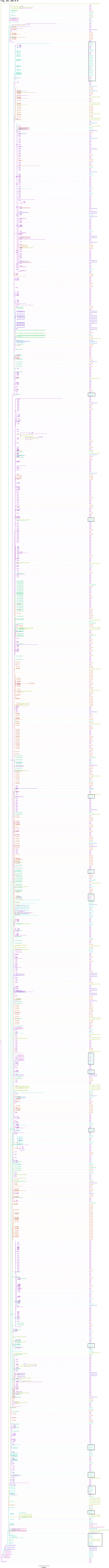

Fig. S7. BA.5.1

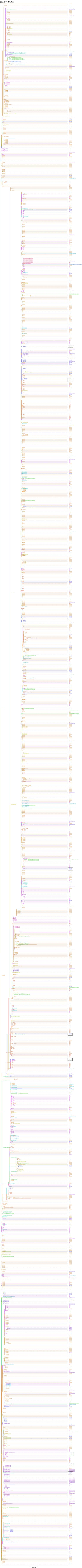

Fig. S8. BA.5.1.1

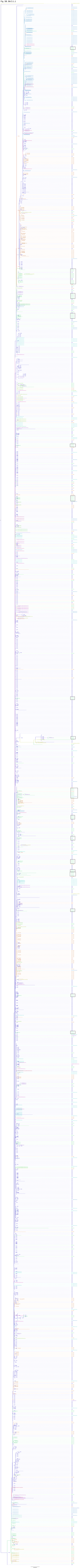

Fig. S9. BA.5.2

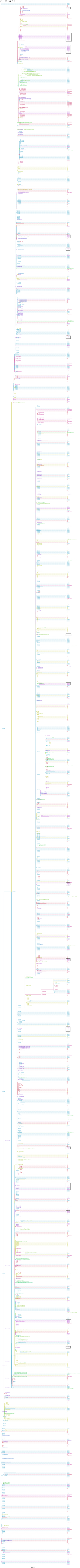

Fig. S10. BA.5.2.1

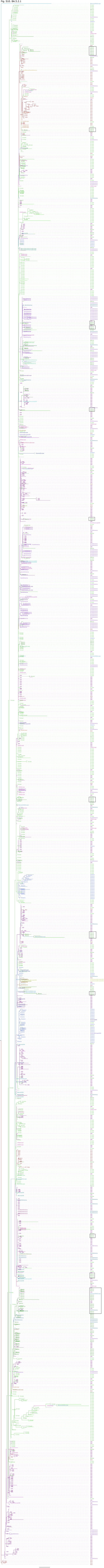

Fig. S11. BF.1

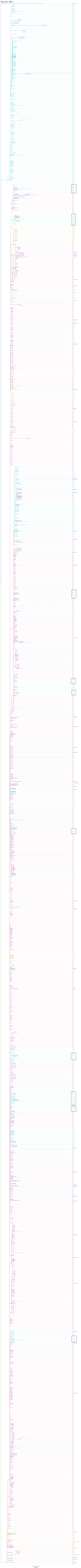

Fig. S12. BE.1

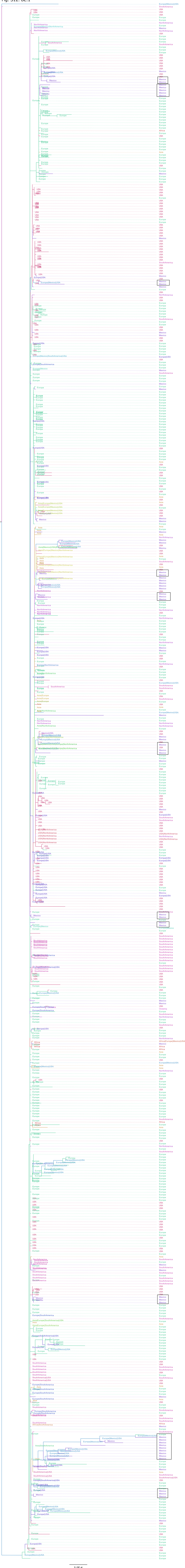

Fig. S13. BE.2

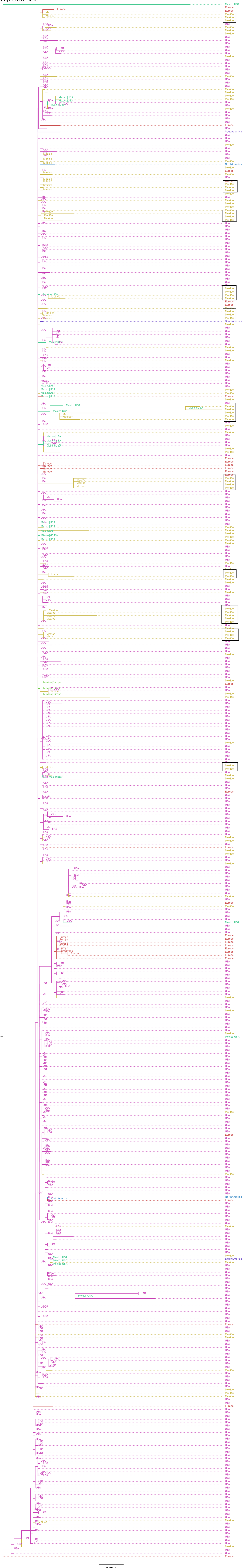

Supplement: Supplementary material 1 [file mgen-9-1120-s001.pdf]
